# Factors Influencing Development and Implementation of Patients' Access to Electronic Health Records—A Comparative Study of Sweden and the Netherlands

**DOI:** 10.3389/fpubh.2021.621210

**Published:** 2021-06-08

**Authors:** Charlotte D. Cijvat, Ronald Cornet, Maria Hägglund

**Affiliations:** ^1^Amsterdam Public Health, Medical Informatics, Amsterdam Universitair Medische Centra, University of Amsterdam, Amsterdam, Netherlands; ^2^Department of Women's and Children's Health, Uppsala University, Uppsala, Sweden

**Keywords:** patient accessible electronic health record, open notes, patient portal, implementation, consolidated framework for implementation research

## Abstract

**Background:** Patient-accessible electronic health records (PAEHRs) and associated national policies have increasingly been set up over the past two decades. Still little is known about the most effective strategy for developing and implementing PAEHRs. There are many stakeholders to take into account, and previous research focuses on the viewpoints of patients and healthcare professionals. Many known barriers and challenges could be solved by involving end-users in the development and implementation process. This study therefore compares barriers and facilitators for PAEHR development and implementation, both general and specific for patient involvement, that were present in Sweden and the Netherlands.

**Methods:** There were a total of 14 semi-structured interviews with 16 key informants from both countries, on which content analysis was performed. The Consolidated Framework for Implementation Research was used to guide both the construction of the interview guides and the content analysis.

**Outcomes:** The main barriers present in both countries are resistance from healthcare professionals and technical barriers regarding electronic health record systems and vendors. Facilitators varied across the two contexts, where the national infrastructure and program management were highlighted as facilitators in Sweden and stakeholder engagement (including patients and healthcare professionals) was described as a facilitator in both contexts. Strong leadership was also described as a critical success factor, especially when faced with healthcare professional resistance.

**Conclusion:** Most of the major barriers and facilitators from both countries are covered in existing literature. This study, however, identified factors that can be seen as more practical and that would not have arisen from interviews with patients or physicians. Recommendations for policymakers include keeping the mentioned barriers in mind from the start of development and paving the way for facilitators, mainly strict policies, learning from peer implementers, and patient involvement, when possible. Implementers should focus on strong decision-making and project management and on preparing the healthcare organization for the PAEHR.

## Introduction

Over the past two decades, advancements in law, technology, and policy have stimulated the implementation of patient-accessible electronic health records (PAEHRs) (1). These systems, set up by either healthcare providers or governments, allow patients to access their medical data whenever they want. PAEHRs can be designed and implemented in different ways, including logging in to a web-accessible portal to read the EHR information and potentially downloading this information into a personal health record (PHR). In the USA, a distinction is often made between access to, e.g., lab results and access to the actual notes in the record, with the latter referred to as *open notes* (2, 3). The concept PAEHR, on the other hand, refers to patients' access to all the content in the electronic health record (EHR) (1), including, but not limited to, clinical notes. Providing access to medical data potentially improves patient empowerment, leading to less consultations and more efficient healthcare provision, thus lowering healthcare expenditure and resulting in better health outcomes (4). Since these benefits can occur not only on an individual healthcare provider level but also for the whole healthcare system, many countries take a national approach to either developing national PAEHRs or creating national policies for implementation (5).

Even though more and more PAEHRs have been implemented, still little is known about the most effective strategy for developing and implementing PAEHRs and associated policy. Implementation can be defined as “the constellation of processes intended to get an intervention into use within an organization; it is the means by which an intervention is assimilated into an organization” (6). In the case of PAEHRs, there are many human, organizational, and technological factors that can complicate these processes (7). There are, for example, many different stakeholders affected by PAEHRs, all with different and sometimes contradictory concerns, incentives, or demands (8, 9). Existing literature mainly focuses on individual cases and on the viewpoints of patients (10, 11) or healthcare professionals (HCPs) (12) rather than the people responsible for developing or implementing PAEHR policy (13). Progress, internationally, has been slow due to legal constraints, technical challenges, and concerns or resistance among HCPs (14). Low rates of adoption among patients have also been a problem in some areas (14). Nonetheless, research evidence reports positive outcomes among patients accessing their records (3, 10), and the concerns expressed by HCPs have not been realized. Patients who read their notes report understanding their care plans better (3), feeling more in control of their care (3, 10), doing a better job taking their medications (15), improved communication with and trust in their clinicians (10, 15), and improved patient safety (16). Studies focusing on implementation barriers stress the importance of involving end-users' viewpoints—in this case, the patients—in the development and implementation (4, 17, 18). For example, patient-reported barriers for PAEHR adoption include lack of healthcare provider acceptance, endorsement, and promotion of the PAEHR, poor user health literacy, and perceived usability and utility problems (e.g., usefulness of the available information and personalization of the PAEHR).

It has been hypothesized that countries developing PAEHRs and associated policies face similar barriers and facilitators, both general and specific, for patient involvement and can improve their existing policies by comparing these factors and learning from each other (19). We have chosen Sweden and the Netherlands as two contexts to explore and compare in this study. It is expected that the outcomes of this study can help Sweden, the Netherlands, and potentially other countries with similar strategies to improve their policies and strategies regarding PAEHR development and implementation. This study aims to:

1. describe and compare barriers and facilitators to implementing PAEHRs in Sweden and the Netherlands, and
2. describe and compare different aspects of patient involvement in PAEHR development and implementation processes in Sweden and the Netherlands.

## Methods

To compare the implementation of PAEHRs in Sweden and the Netherlands, we performed semi-structured interviews with key informants in the respective contexts, focusing on barriers and facilitators in the implementation process, as well as on issues specifically related to patient involvement.

### Study Settings

**Figure 1:**
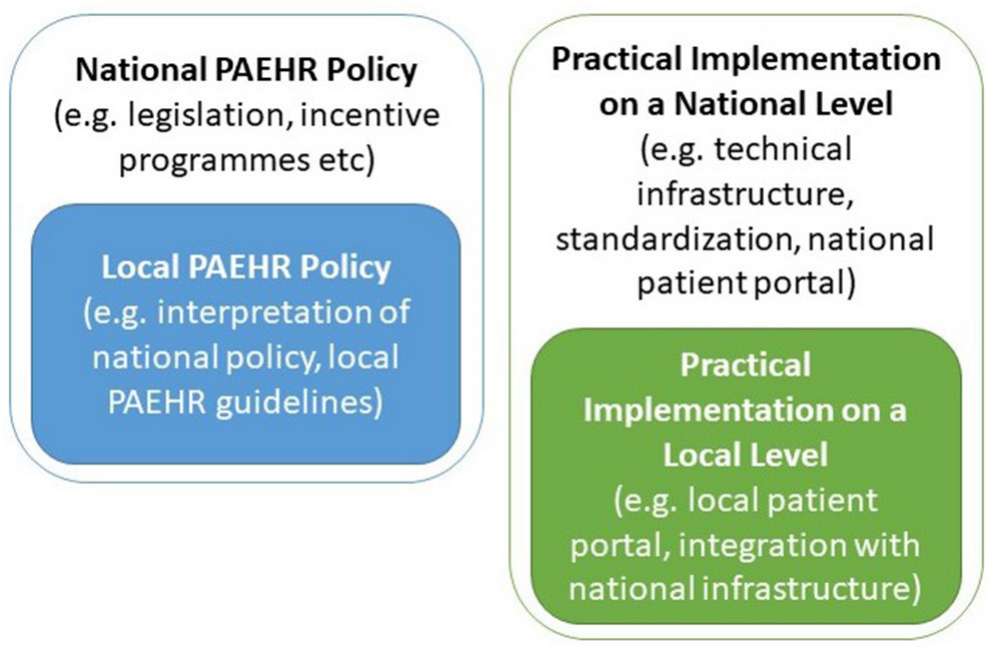
Components of patient-accessible electronic health record implementation.

The study settings presented in Table 1 were used as a guide for identifying the different stakeholders and key informants. Implementation of PAEHRs may consist of several parts; both PAEHR policy and the practical (often both technical and organizational) implementation, which will likely need to take place on both national and local (healthcare provider) levels (Figure 1).

**Table 1:** Overview of healthcare system structures, regulations concerning access to medical data, and existing patient-accessible electronic health record (PAEHR) policies in Sweden and the Netherlands.

	**Sweden**	**The Netherlands**
Number of inhabitants	10 million	17 million
Healthcare system structure	Tax-funded; decentralized: regional governments, 21 regions are responsible for provision of care and may contract both public and private providers	Mandatory private insurance; private care providers deliver care
Laws regarding (digital) access to medical records	All citizens aged 16 and over have a right to directly access different types of health documentation^a^	Patients aged 12 and over have a right to a digital copy of all information included in the record when the data is processed digitally (from July 2020)^b^
PAEHR policy	One PAEHR for all citizens: Journalen, which was developed by Region Uppsala in several projects since 1997 (5). All regions agreed to implement Journalen as part of the national 1177 Healthcare Guide patient portal. It collects data from different EHR systems through a Health Information Exchange (HIE) infrastructure. There are national guidelines, the National Regulatory Framework (NRF), but it is not mandatory to follow	From December 2016 to December 2019, the “Versnellingsprogramma Informatieuitwisseling Patiënt en Professional” (VIPP program) is in operation. It aims to promote general hospitals and other specialist care institutions to provide digital access and improve medication safety. Participation is not mandatory, but a financial incentive is awarded when specific goals are met
Choices in implementation	The first version of the NRF included both mandatory and electable paragraphs. The main decisions for regions were regarding displaying record entries with or without signing by the physician and with or without a 14-day delay (20). In 2016, a new version of the NRF was published with the intention to provide patients with access to all health and dental care information by 2020	Hospitals can choose which goals regarding patients' access, standardized data capture, and medication verification they want to implement^c^. Providing access is allowed by implementing a patient portal or an upload of EHR information into a PHR. Besides VIPP's goals, choices can be made regarding the history of displayed data, whether information is displayed with or without delay, and the potential functionalities of the portal
Responsible organizations	Inera AB manages the national patient portal 1177.se, including the PAEHR Journalen. It is a company owned by the Swedish regional governments. Regions are responsible for connecting their EHR systems to the national HIE	The Ministry of Health, Welfare, and Sports and the Dutch Hospital Association (NVZ) developed the subsidy programIndividual hospitals carry out the implementation by making arrangements with their EHR system supplier
State of the art	As of March 2018, all regions have connected to the HIE and implemented the PAEHR Journalen. However, some private healthcare providers are still not connected	66 out of 70 non-academic hospitals are participating in VIPP^d^. In December 2017, 30 out of 78 Dutch hospitals had a patient portal with access to medical data^e^

a *https://inera.atlassian.net/wiki/spaces/OIJ/pages/438700782/Nationellt%2Bramverk%2Bf%2Br%2BJournalen*.

b *https://www.rijksoverheid.nl/binaries/rijksoverheid/documenten/brochures/2017/06/01/elektronische-gegevensuitwisseling-in-de-zorg/Wet+elektronische+verwerking+van+gegevens+20170620.pdf*.

c *https://www.vipp-programma.nl/over-vipp/doelstellingen*.

d *VIPP 1 Resultatentabel meting maart 2018: https://drive.google.com/file/d/1qe_owm3U0I2D-osz4Fw413ZlFlXDdxDw/view*.

e *nictiz.siw-ontwikkeling.nl/blog/online-inzage-groeit-door/#*.

Depending on the context, activities may vary between the national and local levels, both with respect to policy and practical implementation. The difference between Sweden and the Netherlands will be highlighted below.

#### The Netherlands

We chose to focus on the “Versnellingsprogramma Informatieuitwisseling Patiënt en Professional” (VIPP program) in the Netherlands. At the time of data collection, the program was in its first phase, which lasted until 2019. It aimed at promoting general hospitals to ensure patients' digital access to medical data and improve medication safety. Participation was not mandatory, but a financial incentive was awarded when hospitals met specific goals of their own selection. Since then, the program has progressed to include other types of healthcare providers and to focus on standardized registration of medical information. Currently, the program is in its fifth phase, which will run until July 2023. Each local healthcare provider is responsible for developing and implementing their online patient portals, and therefore describing them in more detail is difficult.

The VIPP program mainly includes policy on the national level, whereas both policy adaptions, e.g., choice of goals to focus on, and practical implementations, e.g., setting up an online patient portal, occur on the local (hospital/healthcare provider) level.

#### Sweden

In Sweden, 21 regions are responsible for providing healthcare. Although the regions are autonomous and can prioritize which eHealth services to focus on, the national eHealth strategy stipulates that there should be only one online healthcare access point for patients (21). Therefore, a national patient portal, “1177.se,” has been implemented (5). The portal runs on a national health information exchange (HIE) platform (22, 23), and through this infrastructure, data stored in any of the EHR systems used in the 21 regions can be accessed. Authentication with an e-ID gives patients access to a number of administrative services as well as the PAEHR Journalen.

In Sweden, policy is important both on the national and local levels, with a national regulatory framework (NRF) for PAEHR (20, 24), which is adapted on the local/regional level. In contrast to the Netherlands, practical implementation, however, also takes place on both the national and the local level, with the bulk being on the national level with the national HIE platform, the national patient portal, and the national PAEHR. On the local level, practical implementation in Sweden is limited to connecting the local EHR system with the national HIE platform.

Similar to the Swedish context, national patient portals are implemented throughout the Nordic countries, whereas healthcare-provider-specific portals are common beyond the Nordic context, such as in the Netherlands. The Swedish and Dutch cases in this study therefore represent two different approaches that countries take to ensure that citizens have access to their health information. Table 1 gives an overview of the two contexts.

It is important to note that the Dutch VIPP only covers general hospitals and other specialist care institutions, while the Swedish PAEHR “Journalen” can display EHR data from all levels of healthcare. We distinguish developers of national PAEHR policies and solutions from local or regional healthcare providers that implement those.

### Study Participants

We purposefully chose key informants in both contexts that could provide insights into the research questions. Personal and professional networks were used in Sweden, and in the Netherlands, the participants were recruited through the VIPP organization. An element of snowball sampling was also applied, where the initially identified key informants recommended others. In total, 16 key informants chose to participate in the study (see Table 2 for a description of the respondents' roles).

**Table 2:** Overview of the interview respondents.

**Interview**	**Respondent**	**Organization**	**Role related to patient-accessible electronic health record**
**Sweden**			
1	1	Inera	Head of Journalen
2	2	Region Uppsala	Project manager/coordinator in several projects of Journalen development and implementation
3	3	Region Uppsala	Medical expert involved in several projects of Journalen development and implementation
4^a^	4	Region 2	Project leader of Journalen implementation
5^a^	5	Region 2Private caregiver	Member of steering committee for Journalen implementation Chief medical informatics officer
6^a^	6, 7, 8	Region 2	Participant in central work of Journalen implementation
7	9	Region 3	Project leader for healthcare IT implementation
8	10	University	Researcher
**The Netherlands**			
9	11	IT advising companyHospital 1	Senior advisor Project leader
10	12	Hospital 2	Project manager
11	13	Hospital 3	Project leader/advisor
12	14	Hospital 4	Project leader
13	15	VIPP programDutch hospital organization	Project leader Senior policy advisor
14	16	Patient federation	Policy advisor

a *The interview was performed via email*.

In Sweden, the original developers of the PAEHR Journalen from Region Uppsala, the current responsible organization Inera AB, and other regions and healthcare providers that have implemented the PAEHR Journalen were approached. In the Netherlands, we interviewed the decision-makers of the VIPP program and experienced experts of implementing PAEHRs according to VIPP. In this study, we focused on the project managers' and implementers' perspective, not those of patients and HCPs which have been covered more extensively in the literature already.

### Data Collection

Semi-structured interviews were conducted between March and May 2018 *via* Skype or phone, where possible. All interviews with Dutch respondents were conducted in Dutch. All interviews were performed by the first author, who speaks Dutch but not Swedish. Some Swedish respondents were reluctant to conduct the interviews in English and were offered to (iteratively) answer questions via email in Swedish. Their answers were translated into English with the help of a native Swedish speaker. The remainder of the Swedish respondents participated in English. This applied to interview numbers 4–6 (marked in Table 2) with five respondents.

Semi-structured interview guides were established for each respondent separately based on their role and the context they practice in. The interview guide revolved around the following topics:

- factors affecting the implementation: perceived barriers and facilitators, and
- patient involvement: necessity, ideal execution, execution in reality, outcomes, and consequences

**Figure 2:**
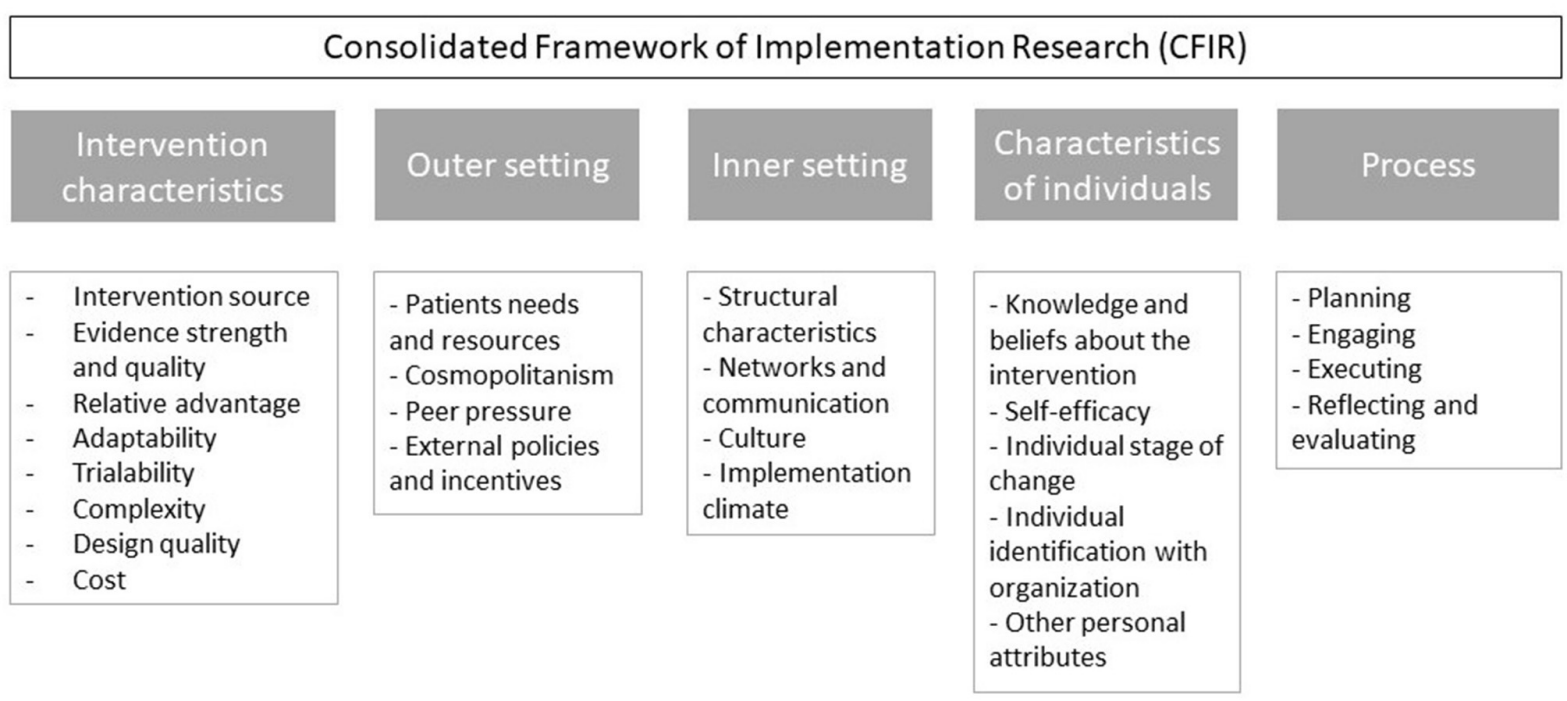
Overview of the consolidated framework for implementation research.

The interview guides were based on the Consolidated Framework for Implementation Research (CFIR), which identifies five dimensions that are essential to implement an intervention (6). An overview of the CFIR dimensions and sub-constructs is given in Figure 2.

The framework further provides sub-constructs and related questions for each of the dimensions (6). The structure of the interview guides was determined by the sub-constructs that were deemed relevant for each type of respondent, i.e., respondents on the national or local level for both Sweden and the Netherlands.

To inquire about the factors affecting PAEHR implementation, the following selection of (sub)constructs was used: the strength and quality of evidence for the intervention, the external policies and incentives, the implementation climate, and all stages of the process (planning, engaging, executing, reflecting, and evaluating). The constructs that patient needs and resources and tension for change were used to assess the involvement of patients.

In addition, several other sub-constructs were selected for specific respondent types, such as peer pressure among Swedish regions. The questions proposed by the CFIR framework were adapted to each individual respondent's specific context. The interview guides were further improved iteratively after each interview to ensure that all relevant aspects were covered.

All participants were informed about the goals and risks of the study. If applicable, audio was recorded, and notes were kept. The interviews were transcribed as soon as possible using intelligent verbatim transcription. While listening to the recordings, transcripts were shortened and edited for the sake of clarity before analysis.

### Analysis

The interviews were analyzed by means of content analysis according to Taylor-Powell and Renner (25). The selected CFIR constructs and sub-constructs formed the basis of the codes with which relevant passages from the edited transcripts were categorized. All passages were provided with a condensed meaning unit in English. Related passages and condensed meaning units were collected, compared, grouped, and provided with a description.

## Results

The results of our analysis are presented in two sections;

1. analysis of barriers and facilitators to implementation of PAEHRs, and
2. the level and impact of user involvement in the respective setting.

### Factors Affecting the Implementation of PAEHRs

We have identified the main barriers and facilitators to developing and implementing national PAEHR policy as described by the respondents from both countries. Barriers exist both on the national level (Table 3) and on the local implementation level (Table 4). One of the main barriers has been resistance from HCPs on both levels in Sweden and on the local implementation level in the Netherlands. This resistance is very much in line with concerns that have previously been described in the literature; concern that patients will misunderstand, take offense or worry unnecessarily, concern that the workload will increase with patients asking questions, and concern that the records' value will be reduced as it can no longer be used as a tool for professional communication.

“[…] one of the barriers was the healthcare professionals, especially the physicians' professional organization/union. The professional organization was more reluctant to expose it to the patient than the average doctor, is my opinion. Because they wanted to be in control of what the patient read. The legislation says it should not be hidden to the patient, with two exceptions: if someone else is mentioned in the record or if it is proven that the result of a treatment will be worse if the patient is aware of it, then the record can be closed. More or less, I have not found any doctor that has used any of those two reasons for not sending a copy on request of the patient.” (Sweden, local level)

**Table 3:** Barriers on the national level.

**Category**	**Sweden**	**The Netherlands**
Systems and suppliers	Authentication methods	Difficulties in measuring hospitals' progress
Social and organizational	Resistance and fears from physicians	–
Resources	Financing the development of Journalen	–
	Too little time to take precautions for physicians' resistance	
Policies, laws, and regulations	Include electable rules to make progress	Challenging to define goals adequately for desired outcomes
	Electable rules caused confusion and inequality for users	Challenging to estimate reasonable usage percentages
	Giving patients direct online access to the record was illegal when the PAEHR Journalen was first introduced in 2002	Slow development of other national programs
Effects of barriers	Delays	Delays
	Restrictions on information that is displayed	

**Table 4:** Barriers on the local implementation level.

**Category**	**Sweden**	**The Netherlands**
Systems and suppliers	Technical limitations of systems	Limitations in choice and possibilities of systems
	High costs for connecting small EHR systems	Large dependency on software suppliers
	Testing prior to implementation necessary	Alignment of systems necessary but difficult
	Difficult requisites for connecting to the HIE	Systems and suppliers determine achievement of VIPP
Social and organizational	Resistance and fears from physicians	Physicians' reluctancy, resistance, and fears
	Changing HCPs' routines, workflows and attitudes	Changing HCPs' political status and workflow
		Effects on hospitals' culture and work processes
		Fears for patients' confusion, questions, fears
		Gradual implementation necessary to keep physicians on board
Resources	High costs for connecting to the HIE	VIPP requires a lot of human work
	Time-consuming decision making due to flexibility in NRF	Human work leads to high costs
		Too little time to make VIPP's deadlines
Policies, laws, and regulations		Some VIPP goals are difficult to accomplish
		Strict privacy regulations not in patients' interests
		Strict security rules impede user-friendliness
Governance	Gradual approach necessary to get all stakeholders on board	Gradual implementation to keep physicians on board
	Flexibility in choosing EHR systems in some counties but only one supported	VIPP has no or low priority
		Cooperation between stakeholders necessary
Effects of barriers	Delays	Delays
	Restrictions on information that is displayed	High costs for implementing systems and VIPP
		Too little time to create support from staff
		Low user-friendliness and usage
		VIPP has low priority

Regarding policy, both countries had some challenges on the national level. In Sweden, it was difficult to agree on a NRF, and the first version had several “electable rules,” i.e., rules where each region or local healthcare provider had to choose how to implement (20). One could, e.g., choose which types of information to release to patients (notes, lab results, referrals, *etc*.), also whether to give immediate access or have a 2-week waiting period, and whether to release only signed/validated information or not. This, of course, led to a fragmentation of the otherwise national PAEHR, where it was difficult for patients to understand why they could see lab results from Region A but not from Region B. In the Netherlands, all practical implementation took place at the local level, but the VIPP program provided an important incentive. Here it was, however, difficult to both define goals and measure the hospitals' progress toward these goals.

“So, at the very last moment we thought, oh, something with an auditor and a manual is also still necessary. You can see that hospitals want to know everything down to the decimal point: what is meant with this, what do you want to achieve with that? […] You see that such a manual in auditor language is difficult to interpret for hospitals.” (the Netherlands, national level)

“I wonder whether the VIPP program actually delivers what it aims to deliver. This is mainly due to the audit questions, which are very technically structured: is this available, do you offer this…? I think that the reality in the workplace has not been looked at very carefully, whether these audit standards match reality. And there is a lot of confusion.” (the Netherlands, local level)

Implementers from both countries faced technical barriers when implementing PAEHRs. In Sweden, the first pilot projects struggled to find secure authentication methods, but in later implementations, the technical challenges related mainly to connecting the EHR systems to the national HIE platform:

“The first barriers were strictly technical, making sure that we had the right protocols from the supplier of the EHR and making sure that everything worked fine in that integration.” (Sweden, local level)

Connecting to the national HIE platform was not only described as a technical challenge; it could also be quite costly, which kept smaller private healthcare providers from connecting:

“[…] healthcare providers in our region can use whatever EHR system they want. It is expensive to make changes in the systems unless they are big. That's why there are healthcare providers who are still not connected. All hospitals and most health centers are connected.” (Sweden, local level)

Dutch implementers were dependent on their IT suppliers for implementing a successful PAEHR and achieving the VIPP goals:

“At the moment, we have a technological status in hospitals in the Netherlands; we have two suppliers who, kind of, wield the scepter, and we are therefore largely dependent on the speed at which they develop [the patient portals]. We have limited influence on that.” (the Netherlands, local level)

The Dutch interviews revealed no facilitators on the national level, likely due to the fact that all practical implementations took place at the local hospital level. In Sweden, the National HIE platform as well as the updated, more strict version of the national regulatory framework (24) was described as facilitator (Table 5).

**Table 5:** Facilitators on the national level.

**Category**	**Sweden**	**The Netherlands**
Systems and suppliers	Use of national HIE created by another project	
	Previous experience and knowledge	
Policies, laws, and regulations	Stricter policy	
Governance	Decision-making on a political level	

On the local implementation level (Table 6), the national infrastructure was also described as a facilitator in Sweden, with the use of national protocols and contracts making it easier for regions and private healthcare providers to integrate. Social aspects were also important Swedish facilitators, where involvement of different stakeholders, learning from peers who were also implementing, and a gradual implementation process were described as beneficial:

“It hasn't been so complicated to implement Journalen because our region was among the last to do it in Sweden. That means we could learn a lot from the experiences of those who had already implemented.” (Sweden, local level)

**Table 6:** Facilitators on the local implementation level.

**Category**	**Sweden**	**The Netherlands**
Systems and suppliers	Use of national protocols and standards	Large EHR system suppliers address security issues
	Reusable contracts and protocols	Portal functionalities existed outside of healthcare
		Think about future development from the start
Social and organizational	Involve HCPs' perspective in decision making	Involve both patients and professionals
	Communicate with stakeholders	NVZ published an analysis of impact on hospitals' work processes
	Gradual implementation	
	Patients can change physicians' behavior if no one else will	
	Ambassadors in healthcare organizations	
Resources	Learn from peers' implementation processes	
	Previous experience and knowledge	
Policies, laws, and regulations	Involve HCPs' perspective in decision making	
Governance	Implementing gradually	Involve both patients and professionals
	Dare to try despite fears from professionals	Strong decision makers
	Central program management	Involve different stakeholders: IT and communication departments, IT suppliers

Involvement of HCPs in the implementation process was described as a facilitator, but there were also some respondents who highlighted the importance of daring to proceed despite resistance from HCPs, indicating that this may be a double-edged sword.

Strong decision-makers and involvement of different stakeholders (including patients and HCPs) were highlighted as facilitators in the Dutch context, too (Table 6):

“Involving HCPs very early and closely in decision-making is extremely important, so you want to treat them very nicely and carefully and never feel like you are making choices over their heads. There is still resistance to portals, and you can take that away by treating them properly. You can never take it away completely, so you have to be persistent. You also need a strong board of directors that make choices or someone else making choices and saying, we are going to do this, even if people are against it. You need that, too; it is also a critical success factor.” (the Netherlands, local level)

“I think the success of this also depends on who gets involved, so if the board of directors finds this very important and gives it a lot of ‘bravado', then it is more likely to lead to success. […] I think that is a success factor that you do not have [at this hospital], which means that the project is also running less than it could. […] Because when the board of directors says: this is what we are going to do—then the specialists and medical managers and business managers will often listen much more carefully. Now, it is me who is always peddling. A hospital is quite hierarchical, which means that it is sometimes necessary for someone to say: this is what we are going to do.” (the Netherlands, local level)

In the Dutch interviews, the EHR vendors' role was more prominent, and their contribution to addressing, e.g., security issues was considered a facilitator:

“Security is often addressed relatively late, like—oh, [the portal] also needs to be safe. That problem is somewhat smaller since we work with the large EHR suppliers because they already have their own ideas on that; they simply offer it in the safest possible way.” (the Netherlands, local level)

Some facilitators and barriers were considered critical. In the Swedish context, the central management of the patient portal and HIE was seen as essential. In the Netherlands, involvement of end-users (both patients and HCPs), implementing toward a clear future goal, involvement of vendors and IT departments, and strong leadership were considered critical success factors. In the Dutch context, the dependence on collaboration with system vendors was also seen as a critical weakness.

### Patient Involvement

The different aspects of patient involvement in PAEHR policy development and implementation in Sweden and the Netherlands are displayed in Table 7 (national level) and Table 8 (local implementation level). In Sweden, patients' wishes and preferences regarding digital access to the EHR were analyzed during the early deployment of the PAEHR Journalen. Regions implementing Journalen also attempted to do so but used fewer means to explore the patients' preferences. In addition, as most decisions relating to the national patient portal and the PAEHR were centralized to the national organization, there was a sense of loss of control on the local/regional level, making the incentives for engaging patients in decision-making limited. In the Netherlands, patient involvement mainly took place at the implementation level, even though hospitals face multiple barriers when doing so. Little to no patient involvement was carried out when developing VIPP.

**Table 7:** Patient involvement on the national level.

**Category**	**Sweden**	**The Netherlands**
National policy's intended benefits	Improve patient empowerment	Improve medication safety
	Improve efficiency of medical services	Provide information access for patients
	Digital “self-service” for patients	
Patient-centeredness of national policy	NRF version 1 and 2 have the same goals	VIPP is developed for patients
	Access needs to be improved for persons aged 13–15	
Methods/tools for patient involvement	Workshops with patients and caregivers	
	User surveys	
	Collecting feedback	
Gained understanding and insights	Users want direct access to signed and unsigned notes, preferably in the professionals' language	
	Users want to make their own decision about viewing the information with or without delay	
	Less negative outcomes for patients than expected	
Challenges	Make compromises between patients' and HCPs' wishes	

**Table 8:** Patient involvement on the local implementation level.

**Category**	**Sweden**	**The Netherlands**
Importance or necessity	Leads to better care provision	Added value for patients and their treatment
		To accomplish VIPP
Patient-centeredness of national policy	NRF version 2 is more transparent and supporting to patients than version 1	VIPP is developed for professionals
		Patients will benefit from the information that is displayed
Methods/tools for patient involvement	User surveys	(Online) panels, focus groups
	Collecting feedback	User surveys
	Patient advisory board	Collecting feedback
	Research	Client council members
	Assumptions from HCPs	Research and publications
Gained understanding and insights		Wishes, needs, complaints and questions
		Insights into desired future functionalities
		Debates between client council members and medical staff, which sometimes lead to more support from staff for the patients' wishes
Challenges	Make compromises between patients' and HCPs' wishes	Not possible to combine with VIPP and its technical focus
	Few decisions to involve patients in	Find enough users that are willing and able to participate, have the right mindset and are representative for the hospital's patient population
		Did not get enough feedback from patients
Lack of resources	Patients had too little knowledge or experience to involve in the development process	Not enough time or other resources for patient involvement
		Technically not possible to meet the patients' wishes and requirements
		Too many different wishes and requirements to take into account

## Discussion

### Principal Findings

Swedish and Dutch developers and implementers of national PAEHR policy have reported on many different barriers, facilitators, critical success factors, and aspects of patient involvement. These outcomes are compared and linked to existing literature in order to interpret them and give recommendations.

#### Barriers, Facilitators, and Critical Success Factors

The main barrier that both countries face is resistance from HCPs, which is confirmed in many other studies (4, 9, 12, 14, 26). Even though the resistance presents itself on different levels in Sweden and the Netherlands, the approaches for dealing with it are similar. Both countries felt the need to involve the professionals' viewpoint in decision-making. Even if this compromised the patients' preferences, it was a necessary step to make progress. Multiple Dutch stakeholders perceive involving both the patient and professional perspective as a critical success factor because it is not likely that professionals will be enthusiastic about and use a tool that is created for patients alone and *vice versa*.

The importance of involving patients' (17, 27–29) and HCPs' perspectives (26, 28) in the implementation processes is supported by many studies. Besides stakeholder involvement, the necessity for strong leadership and a gradual approach in implementing were expressed in both countries. Strong leadership is recognized as an important factor for implementation success (27, 30), while a gradual implementation strategy can be seen as a way of reducing the HCPs' resistance, for instance, by implementing functionalities or types of information one at a time. However, gradual implementation was viewed as both a barrier and facilitator in Sweden, whereas the delays it caused in the implementation process was only seen as a barrier for Dutch respondents. Another potential solution to HCP resistance that was mentioned in both countries was enforcing stricter rules or regulations regarding the PAEHR. This is being done, e.g., in the USA, where from April 5, 2021, new federal laws will mandate that providers must extend open notes to all patients, with a few permitted exemptions (31). In Sweden, this could have reduced the inequality in access between regions and let the PAEHR Journalen meet citizens' preferences better. Even though providing digital access is mandatory in the Netherlands from 2020, stricter regulations could have saved time and money that is now needed for convincing HCPs.

Even though technical barriers are reported less often in literature (32, 33), they are present on the implementation level in both countries. In the Netherlands, hospitals are dependent on their software vendors for implementing a successful PAEHR and achieving the VIPP goals. Swedish healthcare providers are faced with high integration costs when connecting EHR systems to the HIE. In addition, it is not always possible to show all types of information that are desired due to technical limitations of the EHR systems that are connected to the HIE. Swedish implementers, however, mentioned that sometimes the reuse of protocols and contracts from other regions or healthcare providers is possible, which facilitates the integration. Regions also have the possibility to take note of and learn from social aspects of implementing the PAEHR Journalen in other regions. Dutch hospitals sometimes do the latter as well, whereas technical collaboration appears limited. Even though the majority of Dutch hospitals use one of two large EHR systems and their corresponding patient portal, the implementation of VIPP in practice is dependent on more factors such as the pharmacy's medication system. Learning from peers' implementations seems to be very valuable, but has not been mentioned in literature, as a factor playing a role in the implementation process. Concerns about privacy, security, and authentication are recurrent barriers in literature (4, 14, 17, 27, 29), which are surprisingly not mentioned by the respondents in this study. The only barriers related to this domain were about the existing regulations or solutions being too strict and therefore impeding user-friendliness and PAEHR usage.

#### Patient Involvement

The most prominent difference in patient involvement between the two countries is not the reasons for or means of doing it but rather the level on which it is performed. The wishes and preferences of Swedish citizens have been studied and known from the beginning of the PAEHR Journalen's development. Until the new version of the NRF came into place, these preferences had, however, not been taken into account. This is due to the compromises that had to be made between patients' and HCPs' preferences in the development of both the national NRF and regional adaptations of the NRF. The new NRF that was agreed upon in 2016 stipulated that the regions should make all information available without delay by 2020, in accordance with the patients' preferences (34). This is, however, still not the case, and there are so far no consequences for not complying nor incentives to comply. There was little to no patient involvement in developing the Dutch national PAEHR policy, VIPP. Individual hospitals, however, make large efforts in involving users in the development and implementation of their patient portals and even perceive this as a prerequisite for accomplishing VIPP's goals. When doing this, hospitals face different barriers that can be roughly divided into two categories. The first is related to finding the right number of users that are not just willing to participate but also have the right mindset and can together represent the hospital's patient population. The second encompasses barriers that are related to the project itself. These include not having enough time or other resources for patient involvement or not being able to meet the patients' wishes and requirements from either a technical perspective or because there are just too many different wishes and requirements to take into account. Another barrier that cannot be categorized in the previous two groups but that is unforeseen enough to mention is VIPP itself. The program was set up as an incentive to implement (valuable) PAEHRs, but hospitals report that its technical focus leaves no space for patient involvement.

#### Centralized vs. Local Implementation in Practice

**Figure 3:**
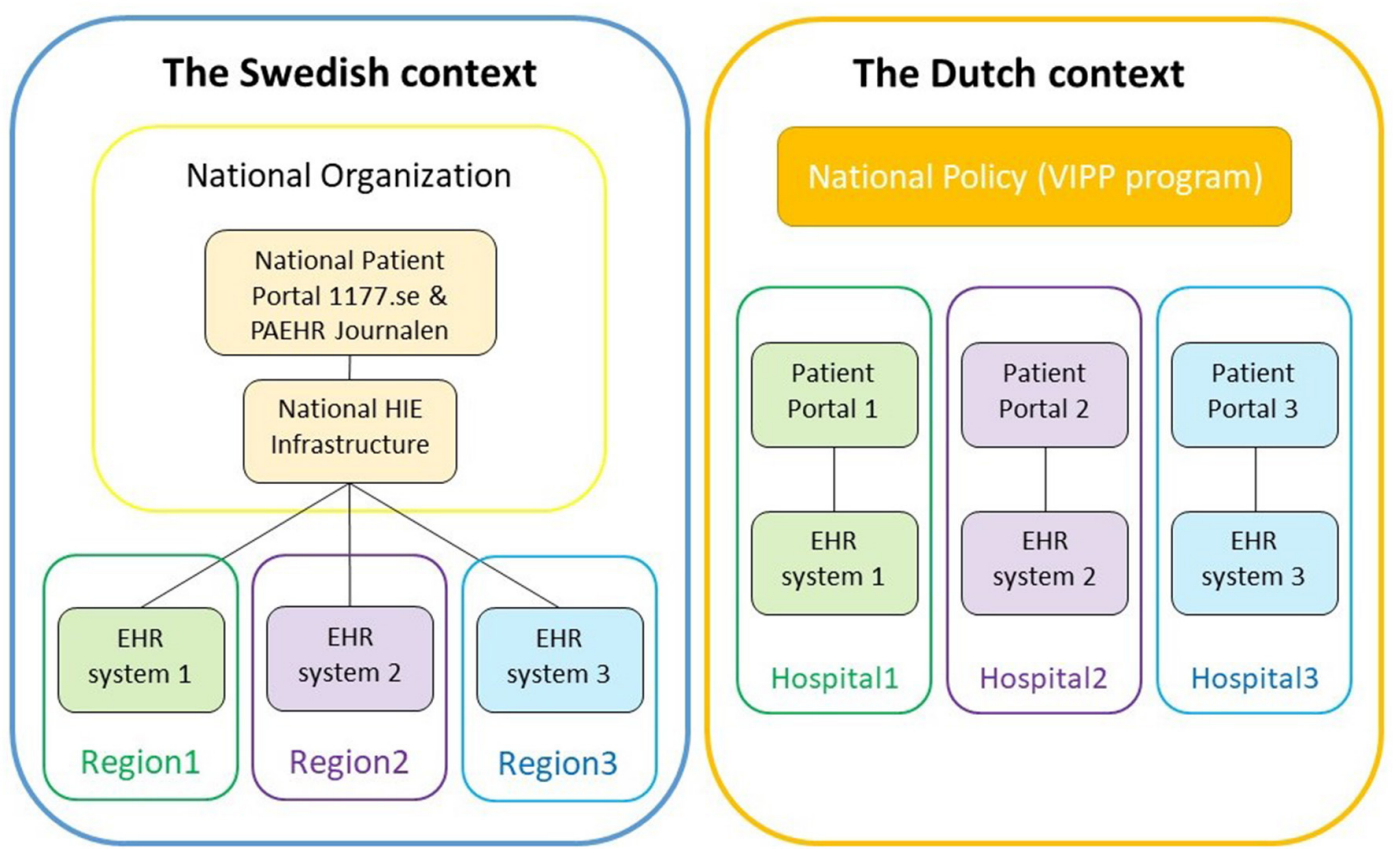
Difference between Sweden's centralized solution and the Netherlands' decentralized.

One of the most important differences between the Swedish and the Dutch contexts is the centralized approach to PAEHR implementation taken in Sweden *vs*. the local implementation in the Netherlands. Figure 3 gives an overview of the difference.

The centralized solution requires, of course, an agreement from all local healthcare providers (in Sweden, the 21 regions and private healthcare providers) to be integrated with the same infrastructure. It has the added benefit for patients in that they only have one access point; even if they move between regions, their data will be in the same patient portal. The distance between the local implementers and the developers of the national patient portal and PAEHR is, however, quite vast, and it is not easy to make local adaptations or pilot new innovative solutions locally.

The decentralized solution, on the other hand, puts all the responsibility of the development of new patient portals and PAEHRs on the local healthcare providers (hospitals in the Netherlands) and requires quite a commitment on their behalf to actually go through with the implementation. As a patient, you may also have to use different portals if receiving care from different healthcare providers. On the other hand, having a closer distance between the local organizations (the hospital and EHR vendor) may facilitate more rapid development and testing of useful functionality, and a variation in PAEHRs could allow for competition and improvements.

### Strengths and Limitations

The strength of this study is that policymakers and implementers were chosen as respondents, while previous studies have mainly focused on the viewpoints of patients and/or HCPs.

Limitations include the sampling method and conduct of the interviews. Even though attempts were made to interview as many respondents as possible, the respondents were sampled though convenience sampling, and it was not investigated how well the implementers represented the whole implementation level in both countries, yet a limited number of people have deeper insights into the implementation process and the individuals interviewed in each context can be considered experts. It is important to note also that the respondents from the implementation levels from both countries were implementing PAEHRs in different settings within healthcare. The interview respondents that implement VIPP all represent hospitals, while Swedish county councils that implement Journalen are responsible for all levels of healthcare. This may, of course, influence the experienced barriers and facilitators, similar to the way other contextual differences do. Some Swedish respondents were offered to answer the interview questions *via* email in Swedish, even though the researcher responsible for data collection was not proficient in this language. The answers were translated together with the last author (who is a native Swedish speaker), yet the email interview format led to less detailed questions and answers than in other interviews. However, the respondents' expertise was considered important, and their answers confirmed results from the more in-depth in-person interviews.

## Conclusion and Recommendations

Most of the major barriers and facilitators that have been mentioned by the PAEHR policy developers and implementers are covered in existing literature, even though previous research generally looked at the viewpoints of patients or HCPs. Our research identified factors that can be seen as more practical and that would not have arisen from interviews with patients or physicians. These include barriers from IT systems and vendors of these systems and the facilitating effect of learning from peers' implementation experiences. While previous literature often mentions concerns about privacy and security as a barrier, this has not been reported by the respondents in this study. We therefore conclude that the factors that affect the PAEHR development and implementation process can differ from the factors that are reported in literature.

We would recommend anyone preparing to implement PAEHRs on a national level or locally in a healthcare organization to consider the factors described in this study when developing and implementing both policy and patient portal/PAEHR. Policy developers can keep the barriers in mind and pave the way for the mentioned facilitators. More specifically, they can consider attaching incentives or penalties to the policy or capturing it in law in order to save resources needed to convince HCPs during implementation. In addition, thoughts can be put into facilitating peer learning among implementers and leaving both room and resources for patient involvement. Implementers should mainly focus on strong leadership, decision-making, and project management, being open to learn from others and allocating resources to possible necessary changes to work practices.

## Data Availability Statement

The datasets presented in this article are not readily available because the data consists of qualitative interview transcripts, and we do not consider this appropriate to make accessible outside the original study. Requests to access the datasets should be directed to maria.hagglund@kbh.uu.se.

## Ethics Statement

Ethical review and approval was not required for the study on human participants in accordance with the local legislation and institutional requirements. The patients/participants provided their written informed consent to participate in this study.

## Author Contributions

CC, RC, and MH participated in the design of the study and analysis of the interviews. CC was responsible for data collection and transcribing of interviews. All the authors participated in the writing of the paper, with CC and MH taking the main responsibility.

## Conflict of Interest

The authors declare that the research was conducted in the absence of any commercial or financial relationships that could be construed as a potential conflict of interest.
